# Supplementary material for: The Impact of Body Mass Index on Latent Tuberculosis Infection: Combined Assessment in People Living with HIV
Source: Pathogens. 2025 Oct 23;14(11):1078. doi: 10.3390/pathogens14111078 (PMC12655615; doi:10.3390/pathogens14111078)
Supplement: Supplementary file 1 [file pathogens-14-01078-s001.zip › pathogens-3892883-supplementary.pdf]

Supplementary

Supplementary Figure S1 EC skin test diameter distribution chart by BMI groups.

Supplementary Figure S2 Correlation analysis of BMI and quantitative value of interferon-γ.

Supplementary Table S1 Demographic characteristics of PLHIV stratified by sites.

Supplementary Table S2 Demographic characteristics of PLHIV stratified by LTBI status and BMI groups.

Supplementary Table S3 Multivariate logistic regression analysis of LTBI stratified by BMI groups.

Supplementary Table S4 Demographic characteristics of PLHIV stratified by CD4+ T cell.

Supplementary Table S5 Demographic characteristics of PLHIV stratified by LTBI status and CD4+ T cell

Supplementary Table S6 Associations of covariates with LTBI from three logistic regression models(LTBI defined by EC skin test).

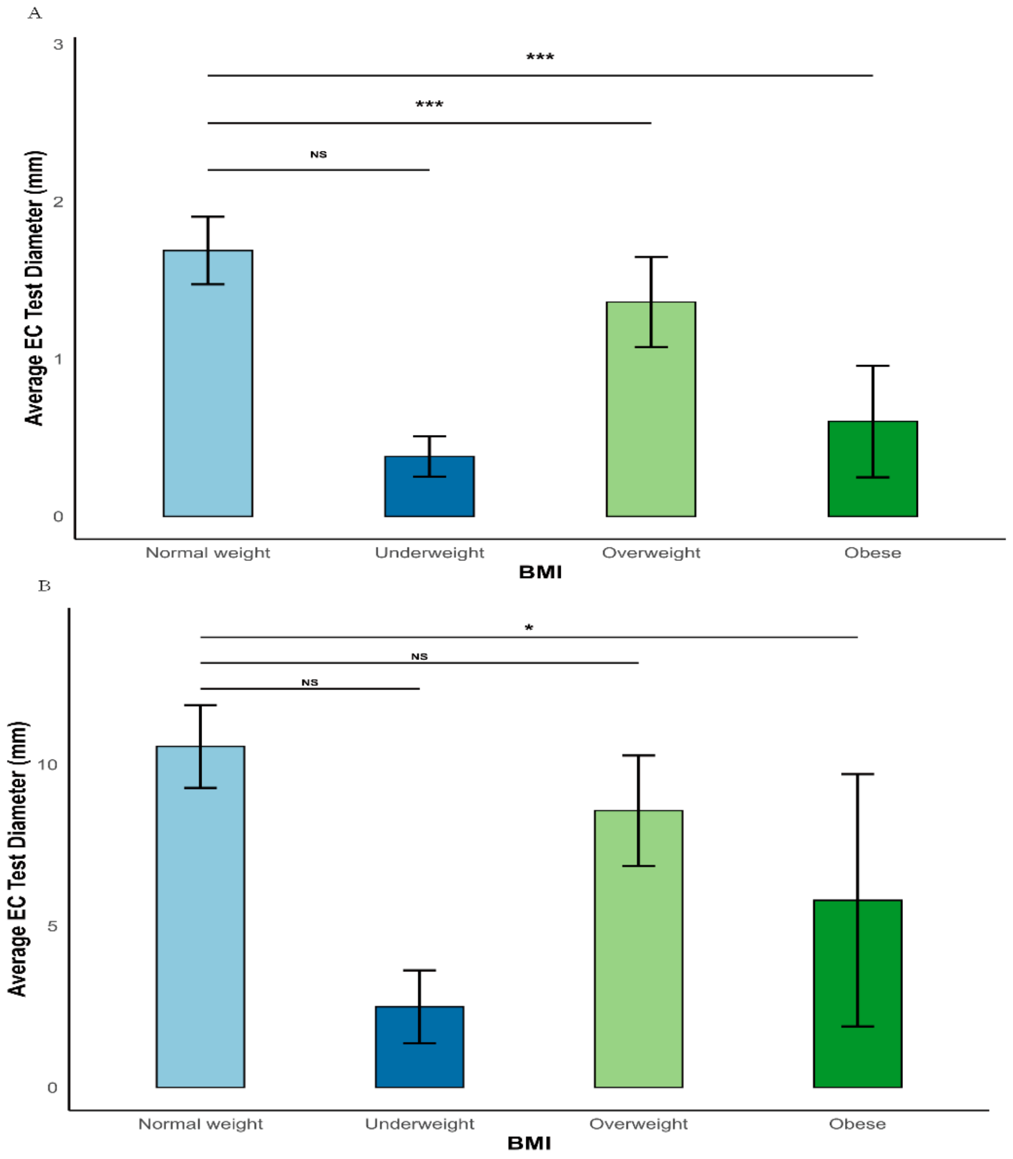

Supplementary Figure S1 EC skin test diameter distribution chart by BMI groups

This figure displays two bar charts (A and B) illustrating the average EC test diameter (in millimeters) across different BMI groups: Normal weight, Underweight, Overweight, and Obese. Chart A: Shows the average EC test diameter in mm for the different BMI groups of all PLHIV. Error bars represent the standard deviation. The asterisks indicate statistically significant differences between groups: '***' denotes $P< 0.001$, and 'NS' indicates no significant difference. Chart B: Presents a similar comparison of the average EC test diameter in mm for the same BMI groups. The error bars again represent standard deviation, and the asterisk indicates statistical significance: '*' denotes $P< 0.05$, and 'NS' indicates no significant difference.

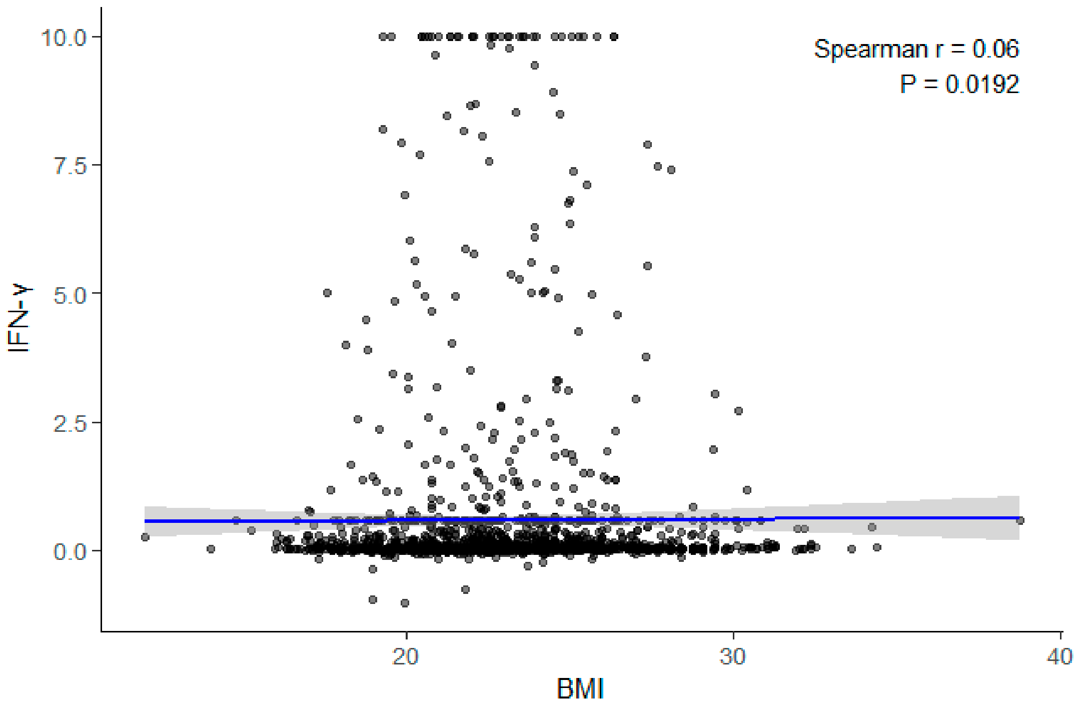

Supplementary Figure S2 Correlation analysis of BMI and quantitative value of interferon-γ

Scatter plot depicting the relationship between BMI on the x-axis and IFN-γ(TB-Nil) values on the Y-axis. Each point represents an individual subject measurement. The blue horizontal line indicates the fitted regression line with 95% confidence intervals (shaded area). Spearman rank correlation analysis revealed a weak positive correlation (r = 0.06, *P* = 0.0192), indicating a statistically significant but modest association between BMI and IFN-γlevels.

Supplementary Table S1 Demographic characteristics of PLHIV stratified by sites.

Variables	Total (n = 1799, %)	Prison (n = 343, %)	Community (n = 1456, %)	Z/χ2	*P*
Age (years)	44.14 ± 13.51	39.15 ± 9.11	45.32 ± 14.10	-10.03	**<.001**
Gender				0.00	0.987
Male	1615 (89.77)	308 (89.80)	1307 (89.77)		
Female	184 (10.23)	35 (10.20)	149 (10.23)		
Ethnic				112.94	**<.001**
Minority	64 (3.56)	45 (13.12)	19 (1.30)		
Han	1735 (96.44)	298 (86.88)	1437 (98.70)		
HIV viral load (copies/mL)	43520.61 ± 281469.61	232.86 ± 2109.75	53718.21 ± 312017.22	-6.54	**<.001**
Education				70.88	**<.001**
Primary school and below	797 (44.30)	218 (63.56)	579 (39.77)		
Middle and high school	421 (23.40)	69 (20.12)	352 (24.18)		
College degree or above	581 (32.30)	56 (16.33)	525 (36.06)		
Occupation				0.45	0.502
Incumbency	763 (42.41)	151 (44.02)	612 (42.03)		
Unemployed or retired	1036 (57.59)	192 (55.98)	844 (57.97)		
Smoke				218.61	**<.001**
No	1115 (61.98)	93 (27.11)	1022 (70.19)		
Yes	684 (38.02)	250 (72.89)	434 (29.81)		
Drink				164.39	**<.001**
No	1283 (71.32)	148 (43.15)	1135 (77.95)		
Yes	516 (28.68)	195 (56.85)	321 (22.05)		
Contact history				55.21	**<.001**
Yes	108 (6.00)	50 (14.58)	58 (3.98)		
No	1691 (94.00)	293 (85.42)	1398 (96.02)		
BCG scars				78.57	**<.001**
No	609 (33.85)	186 (54.23)	423 (29.05)		
Yes	1190 (66.15)	157 (45.77)	1033 (70.95)		
CD4+ T cell (cells/μL)				44.86	**<.001**
≤500	1088 (60.48)	262 (76.38)	826 (56.73)		

Variables	Total (n = 1799, %)	Prison (n = 343, %)	Community (n = 1456, %)	Z/χ2	*P*
＞500	711 (39.52)	81 (23.62)	630 (43.27)		
BMI				15.14	**0.002**
Underweight	106 (5.89)	17 (4.96)	89 (6.11)		
Normal weight	1103 (61.31)	241 (70.26)	862 (59.20)		
Overweight	471 (26.18)	71 (20.70)	400 (27.47)		
Obese	119 (6.61)	14 (4.08)	105 (7.21)		
Diabetes				60.24	**<.001**
No	1431 (79.54)	325 (94.75)	1106 (75.96)		
Yes	368 (20.46)	18 (5.25)	350 (24.04)		
Cardiovascular disease				196.82	**<.001**
No	951 (52.86)	298 (86.88)	653 (44.85)		
Yes	848 (47.14)	45 (13.12)	803 (55.15)		
Silicosis				-	1.000
No	1794 (99.72)	342 (99.71)	1452 (99.73)		
Yes	5 (0.28)	1 (0.29)	4 (0.27)		
Nephropathy				1.72	0.190
No	1736 (96.50)	335 (97.67)	1401 (96.22)		
Yes	63 (3.50)	8 (2.33)	55 (3.78)		
Long-term use of immunosuppressants				47.76	**<.001**
No	1640 (91.16)	280 (81.63)	1360 (93.41)		
Yes	159 (8.84)	63 (18.37)	96 (6.59)		

Supplementary Table S2 Demographic characteristics of PLHIV stratified by LTBI status and BMI groups

Characteristic	Underweight			Normal weight			Overweight			Obese		
	Non-LTBI (n=95, %)	LTBI (n=11, %)	*P*	Non-LTBI (n=946, %)	LTBI (n=157, %)	*P*	Non-LTBI (n=401, %)	LTBI (n=70, %)	*P*	Non-LTBI (n=109, %)	LTBI (n=10, %)	*P*
Age (years)	41.69 ± 17.17	37.82 ± 13.27	0.471	44.22 ± 13.69	43.49 ± 12.33	0.529	44.84 ± 13.00	45.60 ± 12.30	0.649	44.32 ± 12.51	36.40 ± 9.38	0.054
Gender			1			0.606			0.002			0.577
Males	82 (86.32)	10 (90.91)		849 (89.75)	143 (91.08)		368 (91.77)	56 (80.00)		97 (88.99)	10 (100.00)	
Female	13 (13.68)	1 (9.09)		97 (10.25)	14 (8.92)		33 (8.23)	14 (20.00)		12 (11.01)	0 (0.00)	
Ethnic			0.428			<.001			0.001			0.162
Minority	4 (4.21)	1 (9.09)		21 (2.22)	18 (11.46)		10 (2.49)	8 (11.43)		1 (0.92)	1 (10.00)	
Han	91 (95.79)	10 (90.91)		925 (97.78)	139 (88.54)		391 (97.51)	62 (88.57)		108 (99.08)	9 (90.00)	
HIV viral load (copies/mL)	73102.14 ± 574402.31	5174.82 ± 16198.44	0.697	48681.31 ± 295230.66	42666.55 ± 249106.57	0.809	27941.38 ± 163461.47	48836.67 ± 248577.86	0.367	35913.32 ± 196778.72	318.00 ± 652.71	0.570
Education			0.718			<.001			0.011			0.299
Primary school and below	38 (40.00)	6 (54.55)		396 (41.86)	87 (55.41)		166 (41.40)	42 (60.00)		59 (54.13)	3 (30.00)	
Middle and high school	24 (25.26)	2 (18.18)		217 (22.94)	39 (24.84)		97 (24.19)	14 (20.00)		25 (22.94)	3 (30.00)	
College degree or above	33 (34.74)	3 (27.27)		333 (35.20)	31 (19.75)		138 (34.41)	14 (20.00)		25 (22.94)	4 (40.00)	
Occupation			0.007			0.173			0.03			0.452
Incumbency	33 (34.74)	9 (81.82)		397 (41.97)	75 (47.77)		176 (43.89)	21 (30.00)		46 (42.20)	6 (60.00)	
Unemployed or retired	62 (65.26)	2 (18.18)		549 (58.03)	82 (52.23)		225 (56.11)	49 (70.00)		63 (57.80)	4 (40.00)	

Smoke			0.929			<.001			0.018			1
No	63 (66.32)	8 (72.73)		595 (62.90)	76 (48.41)		265 (66.08)	36 (51.43)		66 (60.55)	6 (60.00)	
Yes	32 (33.68)	3 (27.27)		351 (37.10)	81 (51.59)		136 (33.92)	34 (48.57)		43 (39.45)	4 (40.00)	
Drink			0.716			0.034			0.218			1
No	68 (71.58)	9 (81.82)		692 (73.15)	102 (64.97)		287 (71.57)	45 (64.29)		73 (66.97)	7 (70.00)	
Yes	27 (28.42)	2 (18.18)		254 (26.85)	55 (35.03)		114 (28.43)	25 (35.71)		36 (33.03)	3 (30.00)	
Contact history			0.117			<.001			0.024			1
Yes	4 (4.21)	2 (18.18)		43 (4.55)	23 (14.65)		20 (4.99)	9 (12.86)		7 (6.42)	0 (0.00)	
No	91 (95.79)	9 (81.82)		903 (95.45)	134 (85.35)		381 (95.01)	61 (87.14)		102 (93.58)	10 (100.00)	
BCG scars			0.131			0.001			0.106			1
No	26 (27.37)	6 (54.55)		294 (31.08)	69 (43.95)		132 (32.92)	30 (42.86)		38 (34.86)	3 (30.00)	
Yes	69 (72.63)	5 (45.45)		652 (68.92)	88 (56.05)		269 (67.08)	40 (57.14)		71 (65.14)	7 (70.00)	
CD4+ T cell (cells/μL)			0.207			0.166			0.586			0.056
≤500	64 (67.37)	10 (90.91)		597 (63.11)	90 (57.32)		226 (56.36)	37 (52.86)		62 (56.88)	2 (20.00)	
＞500	31 (32.63)	1 (9.09)		349 (36.89)	67 (42.68)		175 (43.64)	33 (47.14)		47 (43.12)	8 (80.00)	
Source			0.523			<.001			<.001			0.74
Prison	14 (14.74)	3 (27.27)		177 (18.71)	64 (40.76)		48 (11.97)	23 (32.86)		12 (11.01)	2 (20.00)	
Community	81 (85.26)	8 (72.73)		769 (81.29)	93 (59.24)		353 (88.03)	47 (67.14)		97 (88.99)	8 (80.00)	
Diabetes			0.455			0.487			0.32			1
No	82 (86.32)	8 (72.73)		761 (80.44)	130 (82.80)		311 (77.56)	58 (82.86)		74 (67.89)	7 (70.00)	
Yes	13 (13.68)	3 (27.27)		185 (19.56)	27 (17.20)		90 (22.44)	12 (17.14)		35 (32.11)	3 (30.00)	
Cardiovascular disease			0.448			0.651			0.979			1
No	49 (51.58)	7 (63.64)		530 (56.03)	91 (57.96)		184 (45.89)	32 (45.71)		53 (48.62)	5 (50.00)	
Yes	46 (48.42)	4 (36.36)		416 (43.97)	66 (42.04)		217 (54.11)	38 (54.29)		56 (51.38)	5 (50.00)	
Silicosis			0.198			0.369			/			/

No	94 (98.95)	10 (90.91)		944 (99.79)	156 (99.36)		401(100%)	7(100%)		109(100%)	10(100%)	
Yes	1 (1.05)	1 (9.09)		2 (0.21)	1 (0.64)		/	/		/	/	
Nephropathy			0.359			0.319			0.258			1
No	92 (96.84)	10 (90.91)		906 (95.77)	153 (97.45)		388 (96.76)	70 (100.00)		107 (98.17)	10 (100.00)	
Yes	3 (3.16)	1 (9.09)		40 (4.23)	4 (2.55)		13 (3.24)	0 (0.00)		2 (1.83)	0 (0.00)	
Long-term use of immunosuppressants			0.593			0.13			0.918			1
No	86 (90.53)	11 (100.00)		842 (89.01)	146 (92.99)		377 (94.01)	65 (92.86)		103 (94.50)	10 (100.00)	
Yes	9 (9.47)	0 (0.00)		104 (10.99)	11 (7.01)		24 (5.99)	5 (7.14)		6 (5.50)	0 (0.00)	

Supplementary Table S3 Multivariate logistic regression analysis of LTBI stratified by BMI groups

Characteristic	N (%)	cOR, 95CI, *P*	aOR, 95CI, *P*
**Underweight (BMI: <18.5)**			
Occupation			
Incumbency	42 (39.62)		/
Unemployed or retired	64 (60.38)	0.12 (0.02–0.58), 0.008	/
**Normal weight (BMI: 18.5–**			
Education			
Primary school and below	483 (43.79)	1.00 (Reference)	1.00 (Reference)
Middle and high school	256 (23.21)	0.82 (0.54–1.24), 0.34	1.00 (0.65–1.53), 0.994
College degree or above	364 (33.00)	0.42 (0.27–0.65), <.001	0.59 (0.37–0.94), 0.025
Ethnic			
Minority	39 (3.54)	1.00 (Reference)	1.00 (Reference)
Han	1064 (96.46)	0.18 (0.09–0.34), <.001	0.38 (0.19–0.79), 0.01
Smoke			
No	671 (60.83)	1.00 (Reference)	1.00 (Reference)
Yes	432 (39.17)	1.81 (1.29–2.54), <.001	1.22 (0.82–1.82), 0.335
Drink			
No	794 (71.99)	1.00 (Reference)	1.00 (Reference)
Yes	309 (28.01)	1.47 (1.03–2.10), 0.035	0.89 (0.58–1.37), 0.609
Contact history			
Yes	66 (5.98)	1.00 (Reference)	1.00 (Reference)
No	1037 (94.02)	0.28 (0.16–0.48), <.001	0.38 (0.21–0.68), 0.001
BCG scars			
No	363 (32.91)	1.00 (Reference)	1.00 (Reference)
Yes	740 (67.09)	0.58 (0.41–0.81), 0.002	0.79 (0.55–1.15), 0.218
Source			
Prison	241 (21.85)	1.00 (Reference)	1.00 (Reference)
Community	862 (78.15)	0.33 (0.23–0.48), <.001	0.52 (0.33–0.80), 0.003
**Overweight (BMI: 25–28)**			
Ethnic			
Minority	18 (3.82)	1.00 (Reference)	1.00 (Reference)
Han	453 (96.18)	0.20 (0.08–0.52), 0.001	0.40 (0.13–1.23), 0.11
Education			
Primary school and below	208 (44.16)	1.00 (Reference)	1.00 (Reference)
Middle and high school	111 (23.57)	0.57 (0.30–1.10), 0.093	0.80 (0.39–1.63), 0.542
College degree or above	152 (32.27)	0.40 (0.21–0.76), 0.006	0.62 (0.30–1.27), 0.192
Occupation			

Incumbency	197 (41.83)	1.00 (Reference)	1.00 (Reference)
Unemployed or retired	274 (58.17)	1.83 (1.06–3.16), 0.031	2.08 (1.16–3.72), 0.014
Smoke			
No	301 (63.91)	1.00 (Reference)	1.00 (Reference)
Yes	170 (36.09)	1.84 (1.10–3.07), 0.02	1.22 (0.65–2.28), 0.536
Contact history			
Yes	29 (6.16)	1.00 (Reference)	1.00 (Reference)
No	442 (93.84)	0.36 (0.15–0.82), 0.015	0.45 (0.18–1.11), 0.083
Source			
Prison	71 (15.07)	1.00 (Reference)	1.00 (Reference)
Community	400 (84.93)	0.28 (0.16–0.50), <.001	0.40 (0.19–0.83), 0.014
Gender			
Males	424 (90.02)	1.00 (Reference)	1.00 (Reference)
Females	47 (9.98)	2.79 (1.40–5.53), 0.003	2.85 (1.28–6.34), 0.01
**Obese (BMI: >28 kg/m$^{2)}$**			
HIV viral load (copies/mL)	32922.11 ± 188517.20	1.00 (1.00–1.00), 0.249	0.998（0.996–1.000）,0.056
CD4+ T cell (cells/µL)			
≤500	64 (53.78)	1.00 (Reference)	1.00 (Reference)
＞500	55 (46.22)	0.19(0.038–0.99),0.041	0.19(0.04–0.99),0.05
Age (years)	43.66 ± 12.44	1.064(0.997–1.99),0.062	1.09(1.01–1.18),0.03

Multivariable logistic regression models were applied to explore factors associated with LTBI within each BMI group. cOR, crude odds ratio; aOR, adjust odds ratio.

Supplementary Table S4 Demographic characteristics of PLHIV stratified by CD4+ T cell

Variables	Total (n = 1799, %)	CD4+ T cell<500 (n = 1088, %)	CD4+ >500 T cell (n = 711, %)	Z/χ2	*P*
Age (years)	44.14 ± 13.51	45.26 ± 13.53	42.43 ± 13.31	4.37	**<.001**
Gender				1.93	0.165
Males	1615 (89.77)	968 (88.97)	647 (91.00)		
Females	184 (10.23)	120 (11.03)	64 (9.00)		
Ethnic				0.01	0.939
Minority	64 (3.56)	39 (3.58)	25 (3.52)		
Han	1735 (96.44)	1049 (96.42)	686 (96.48)		
HIV viral load	43520.61 ± 281469.61	55347.88 ± 335120.94	25422.07 ± 167773.54	2.50	**0.012**
Education				43.99	**<.001**
Primary school and below	797 (44.30)	550 (50.55)	247 (34.74)		
Middle and high school	421 (23.40)	231 (21.23)	190 (26.72)		
College degree or above	581 (32.30)	307 (28.22)	274 (38.54)		
Occupation				0.06	0.803
Incumbency	763 (42.41)	464 (42.65)	299 (42.05)		
Unemployed or retired	1036 (57.59)	624 (57.35)	412 (57.95)		
Smoke				1.58	0.208
No	1115 (61.98)	687 (63.14)	428 (60.20)		
Yes	684 (38.02)	401 (36.86)	283 (39.80)		

Variables	Total (n = 1799, %)	CD4+ T cell<500 (n = 1088, %)	CD4+ >500 T cell (n = 711, %)	Z/χ2	*P*
Drink				0.19	0.665
No	1283 (71.32)	780 (71.69)	503 (70.75)		
Yes	516 (28.68)	308 (28.31)	208 (29.25)		
Contact history				0.02	0.890
Yes	108 (6.00)	66 (6.07)	42 (5.91)		
No	1691 (94.00)	1022 (93.93)	669 (94.09)		
BCG scars				24.62	**<.001**
No	609 (33.85)	417 (38.33)	192 (27.00)		
Yes	1190 (66.15)	671 (61.67)	519 (73.00)		
Source				44.86	**<.001**
Prison	343 (19.07)	262 (24.08)	81 (11.39)		
Community	1456 (80.93)	826 (75.92)	630 (88.61)		
BMI				11.84	**0.008**
Underweight	106 (5.89)	74 (6.80)	32 (4.50)		
Normal weight	1103 (61.31)	687 (63.14)	416 (58.51)		
Overweight	471 (26.18)	263 (24.17)	208 (29.25)		
Obese	119 (6.61)	64 (5.88)	55 (7.74)		
Diabetes				2.63	0.105
No	1431 (79.54)	879 (80.79)	552 (77.64)		
Yes	368 (20.46)	209 (19.21)	159 (22.36)		

Variables	Total (n = 1799, %)	CD4+ T cell<500 (n = 1088, %)	CD4+ >500 T cell (n = 711, %)	Z/χ2	*P*
Cardiovascular disease				10.70	**0.001**
No	951 (52.86)	609 (55.97)	342 (48.10)		
Yes	848 (47.14)	479 (44.03)	369 (51.90)		
Silicosis				0.19	0.663
No	1794 (99.72)	1084 (99.63)	710 (99.86)		
Yes	5 (0.28)	4 (0.37)	1 (0.14)		
Nephropathy				0.00	0.979
No	1736 (96.50)	1050 (96.51)	686 (96.48)		
Yes	63 (3.50)	38 (3.49)	25 (3.52)		
Long-term use of immunosuppressants				4.05	**0.044**
No	1640 (91.16)	980 (90.07)	660 (92.83)		
Yes	159 (8.84)	108 (9.93)	51 (7.17)		

Supplementary Table S5 Demographic characteristics of PLHIV stratified by LTBI status and CD4+ T cell

Characteristic	CD4+ T cell ≤500			CD4+ T cell ＞500		
	Non-LTBI (n = 949, %)	LTBI (n = 139, %)	*P*	Non-LTBI (n = 949, %)	LTBI (n = 139, %)	*P*
Age (years)	45.37 ± 13.68	44.52 ± 12.45	0.459	42.45 ± 13.50	42.31 ± 12.25	0.923
Gender			0.628			0.426
Males	846 (89.15)	122 (87.77)		550 (91.36)	97 (88.99)	
Females	103 (10.85)	17 (12.23)		52 (8.64)	12 (11.01)	
Ethnic			**<.001**			**0.001**
Minority	21 (2.21)	18 (12.95)		15 (2.49)	10 (9.17)	
Han	928 (97.79)	121 (87.05)		587 (97.51)	99 (90.83)	
HIV viral load (copies/mL)	58951.70 ± 350099.63	30743.40 ± 204728.02	0.354	20217.78 ± 139611.05	54165.01 ± 274948.78	0.052
Education			**<.001**			**0.03**
Primary school and	459 (48.37)	91 (65.47)		200 (33.22)	47 (43.12)	
Middle and high school	205 (21.60)	26 (18.71)		158 (26.25)	32 (29.36)	
College degree or above	285 (30.03)	22 (15.83)		244 (40.53)	30 (27.52)	
Occupation			0.814			0.131
Incumbency	406 (42.78)	58 (41.73)		246 (40.86)	53 (48.62)	
Unemployed or retired	543 (57.22)	81 (58.27)		356 (59.14)	56 (51.38)	
Smoke			**<.001**			0.326
No	622 (65.54)	65 (46.76)		367 (60.96)	61 (55.96)	
Yes	327 (34.46)	74 (53.24)		235 (39.04)	48 (44.04)	
Drink			**0.003**			0.839

No	695 (73.23)	85 (61.15)		425 (70.60)	78 (71.56)	
Yes	254 (26.77)	54 (38.85)		177 (29.40)	31 (28.44)	
Contact history			**<.001**			**0.014**
Yes	44 (4.64)	22 (15.83)		30 (4.98)	12 (11.01)	
No	905 (95.36)	117 (84.17)		572 (95.02)	97 (88.99)	
BCG scars			**0.045**			**0.025**
No	353 (37.20)	64 (46.04)		153 (25.42)	39 (35.78)	
Yes	596 (62.80)	75 (53.96)		449 (74.58)	70 (64.22)	
Source			**<.001**			**<.001**
Prison	194 (20.44)	68 (48.92)		57 (9.47)	24 (22.02)	
Community	755 (79.56)	71 (51.08)		545 (90.53)	85 (77.98)	
Obese			**0.017**			0.866
No	887 (93.47)	137 (98.56)		555 (92.19)	101 (92.66)	
Yes	62 (6.53)	2 (1.44)		47 (7.81)	8 (7.34)	
Diabetes			0.278			0.731
No	762 (80.30)	117 (84.17)		466 (77.41)	86 (78.90)	
Yes	187 (19.70)	22 (15.83)		136 (22.59)	23 (21.10)	
Cardiovascular disease			0.559			0.744
No	528 (55.64)	81 (58.27)		288 (47.84)	54 (49.54)	
Yes	421 (44.36)	58 (41.73)		314 (52.16)	55 (50.46)	
Silicosis			0.102			1
No	947 (99.79)	137 (98.56)		601 (99.83)	109 (100.00)	
Yes	2 (0.21)	2 (1.44)		1 (0.17)	0 (0.00)	
Nephropathy			0.244			0.851

No	913 (96.21)	137 (98.56)		580 (96.35)	106 (97.25)	
Yes	36 (3.79)	2 (1.44)		22 (3.65)	3 (2.75)	
Long-term use of immunosuppressants			0.809			0.052
No	854 (89.99)	126 (90.65)		554 (92.03)	106 (97.25)	
Yes	95 (10.01)	13 (9.35)		48 (7.97)	3 (2.75)	

Supplementary Table S6 Associations of covariates with LTBI from three logistic regression models(LTBI defined by EC skin test).

Variables	Model 1		Model 2		Model 3	
	OR (95%CI)	*P*	OR (95%CI)	*P*	OR (95%CI)	*P*
BMI						
Normal weight	1.00 (Reference)		1.00 (Reference)		1.00 (Reference)	
Underweight	0.38 (0.14–1.06)	0.063	0.43 (0.14–1.27)	0.126	0.43 (0.14–1.30)	0.135
Overweight	0.80 (0.54–1.19)	0.279	0.81 (0.53–1.22)	0.313	0.83 (0.54–1.28)	0.398
Obese	0.17 (0.04–0.68)	**0.013**	0.14 (0.03–0.57)	**0.006**	0.13 (0.03–0.54)	**0.005**
Gender						
Male			1.00 (Reference)		1.00 (Reference)	
Female			0.00 (0.00–Inf)	0.998	0.00 (0.00–Inf)	0.998
Age (years)			0.00 (0.00–Inf)	0.998	0.00 (0.00–Inf)	0.998
Education						
Primary school and below					1.00 (Reference)	
Middle and high school					0.00 (0.00–Inf)	0.997
College degree or above					0.00 (0.00–Inf)	0.997
Occupation						
Incumbency					1.00 (Reference)	
Unemployed or retired					0.00 (0.00–Inf)	0.997
Contact history						

Yes	1.00 (Reference)	
No	0.15 (0.00–6.63)	0.326
Source		
Prison	1.00 (Reference)	
Community	0.22 (0.00–10.19)	0.435
Long-term use of immunosuppressants		
No	1.00 (Reference)	
Yes	0.35 (0.01–11.22)	0.551

Model 1: Crude model. Model 2: Adjusted for age and sex. Model 3: Adjust: Additionally adjusted for Education, Occupation, Contact history, Source, Gender, Age, Long-term use of immunosuppressants.
